# Transgenic Killer Commensal Bacteria as Mucosal Protectants

**DOI:** 10.1100/tsw.2001.32

**Published:** 2001-05-01

**Authors:** Luciano Polonelli

**Affiliations:** Dipartimento di Patologia e Medicina di Laboratorio, Università degli Studi di Parma, Italy

**Keywords:** Killer recombinant antibodies, transgenic commensal bacteris, mucosal immunotherapy

## Abstract

As first line of defense against the majority of infections and primary site for their transmission, mucosal surfaces of the oral cavity and genitourinary, gastrointestinal, and respiratory tracts represent the most suitable sites to deliver protective agents for the prevention of infectious diseases. Mucosal protection is important not only for life threatening diseases but also for opportunistic infections which currently represent a serious burden in terms of morbidity, mortality, and cost of cures. Candida albicans is among the most prevalent causes of mucosal infections not only in immuno- compromised patients, such as HIV-infected subjects who are frequently affected by oral and esophageal candidiasis, but also in otherwise healthy individuals, as in the case of acute vaginitis. Unfortunately, current strategies for mucosal protection against candidiasis are severely limited by the lack of effective vaccines and the relative paucity and toxicity of commercially available antifungal drugs. An additional option has been reported in a recent

An additional option has been reported in a recent issue of *Nature Biotechnology* [1]. By exploiting the idiotypic network, the investigators first generated a recombinant antiidiotypic antibody in the single chain format representing the internal image of a yeast killer toxin characterized by the wide spectrum of antimicrobial activity (KTscFv) [2]. KTscFv, through its ability to mimic the yeast killer toxin activity, exerted a therapeutic effect against *C. albicans* rat vaginal infection. KTscFv, moreover, mimics the functional activity of protective microbicidal antibodies naturally produced during candidal infection in humans [3]. The peculiarity of the new experimental approach is the combination of such exclusive antibiotic KTscFv with a unique delivery system constituted by a naturally transformable commensal bacterium (*Streptococcus gordonii*) which was engineered for secreting or displaying at its surface KTscFv. This delivery system, based on a live recombinant microorganism, was chosen since *S. gordonii* is able to express heterologous proteins efficiently during mucosal colonization and deliver candidate vaccines to a mammalian host for oral and vaginal immunization [4].

Thus, engineered killer commensal bacteria were used for the first time therapeutically for colonizing rat vaginal mucous membranes to eradicate an experimental candidal infection. *In vivo* data showed that recombinant *S. gordonii*, particularly in its KTscFv secreting form, was as effective as a full course of fluconazole in rapidly abating the fungal burden.

One of the main problems connected with the evaluation of the clinical efficacy of antibodies on mucosal surfaces is their high cost and limited production capacity. The short half-life of these molecules demands continuous administration and raises problems of bioavailability. The steady mucosal delivery of microbicidal antibodies permanently expressed by colonizing commensal bacteria is judged of primary importance for a prospective therapeutic intervention. In the study recombinant commensal bacteria persisted to colonize the rat vagina for at least 15 days. This consolidated animal model includes, among its advantages, the requirement of persistent bacterial colonization, and in its estrogen dependence, characteristic invasive hyphal *Candida* growth in loco and vaginal T lymphocyte assets,

Contact info: Phone: 0039 0521 988885; Fax: 0039 0521 993620
E-mail: lucpol@unipr.it
© 2001 with author.

has a strong degree of similarity to human infection [5]. As such, it has been adopted to test the preclinical actrivity of antimycotic and immunotherapeutic agents.

Even though vaginal candidiasis represents an ideal experimental model of mucosal infections, the rationale of the new technology should be applicable to other infections, caused by pathogenic microorganisms proven to be susceptible to the yeast killer toxin, such as *Cryptococcus neoformans*, *Pneumocystis carinii,* and multidrug-resistant strains of *Mycobacterium tuberculosis*, on all mucosal surfaces [6].

The use of other appropriate recombinant mucosal bacteria such as *Lactobacillus* spp. to deliver therapeutic antibodies or peptides may represent an exciting and potentially general strategy for the safe treatment and prevention of different mucosal infections.

In the perspective of clinical trials, however, it remains to be ascertained whether long-term colonization of the human mucosal surfaces with recombinant commensal bacteria is completely safe. Moreover, the means for large scale production and the regulatory pathway for new transgenic commensals will need to be defined.
